# Supplementary material for: Complex Modes of Inheritance in Hereditary Red Blood Cell Disorders: A Case Series Study of 155 Patients
Source: Genes (Basel). 2021 Jun 23;12(7):958. doi: 10.3390/genes12070958 (PMC8304671; doi:10.3390/genes12070958)
Supplement: Supplementary file 1 [file genes-12-00958-s001.zip › genes-1257053-SI.pdf]

Supplementary Data

Complex modes of inheritance in hereditary red blood cell disorders: a case series study of 155 patients

Immacolata Andolfo[1,2,*], Stefania Martone[1,2], Barbara Eleni Rosato[1,2], Roberta Marra[1,2], Antonella Gambale[2,3], Gianluca Forni[4], Valeria Pinto[4], Magnus Göransson[5], Vasiliki Papadopoulou[6], Mathilde Gavillet[6], Mohssen Elalfy[7], Antonella Panarelli[2], Giovanna Tomaiuolo[2,8], Achille Iolascon[1,2] and Roberta Russo[1,2,*]

[1] Dipartimento di Medicina Molecolare e Biotecnologie Mediche, Università degli Studi di Napoli Federico II, Napoli, Italy
[2] CEINGE Biotecnologie Avanzate, Napoli, Italy
[3] Dipartimento Assistenziale di Medicina di Laboratorio (DAIMedLab), UOC Genetica Medica, AOU Federico II, Napoli, Italy
[4] Centro della Microcitemia e delle Anemie Congenite Ente Ospedaliero Ospedali Galliera, Via Volta 6, 16128 Genoa, Italy.
[5] Department of Pediatrics, The Queen Silvia Children's Hospital, Sahlgrenska University Hospital, Gothenburg, Sweden.
[6] Service and Central Laboratory of Hematology, Department of Oncology and Department of Laboratory Medicine and Pathology, Lausanne University Hospital (CHUV), Lausanne, Switzerland.
[7] Thalassemia Center, Faculty of Medicine, Ain Shams University, Cairo, Egypt.
[8] Dipartimento di Ingegneria Chimica, dei Materiali e della Produzione Industriale, Università di Napoli Federico II, Napoli, Italy

Supplemental Data file contains:

- Supplemental Figure S1
- Supplemental Figure S2
- Supplemental Figure S3
- Supplemental Table S1
- Supplemental Table S2

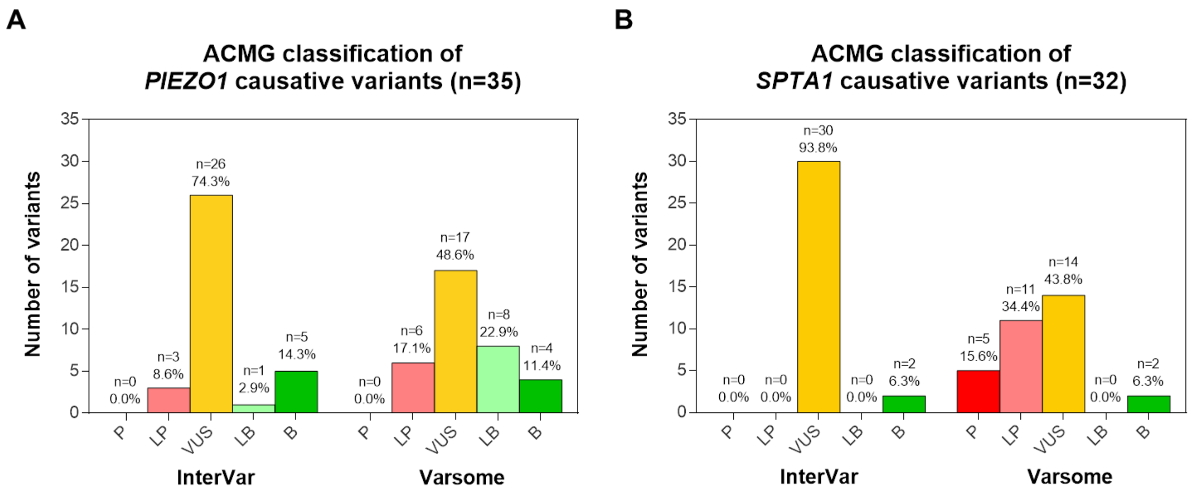

Figure S1. Histogram chart showing the ACMG classification of the *PIEZO1* and *SPTA1* known causative variants. The charts show the number of known causative variants of *PIEZO1* (**A**) and *SPTA1* (**B**) genes classified as P = pathogenic, LP = likely pathogenic, VUS = variants of unknown significance, LB = likely benign, B = benign by two web tools InterVar (http://wintervar.wglab.org/) and Varsome (https://varsome.com/). The two tools were used for clinical interpretation of the known causative variants of both the genes, following the American College of Medical Genetics and Genomics (ACMG) and the Association for Molecular Pathology (AMP) 2015 guidelines.

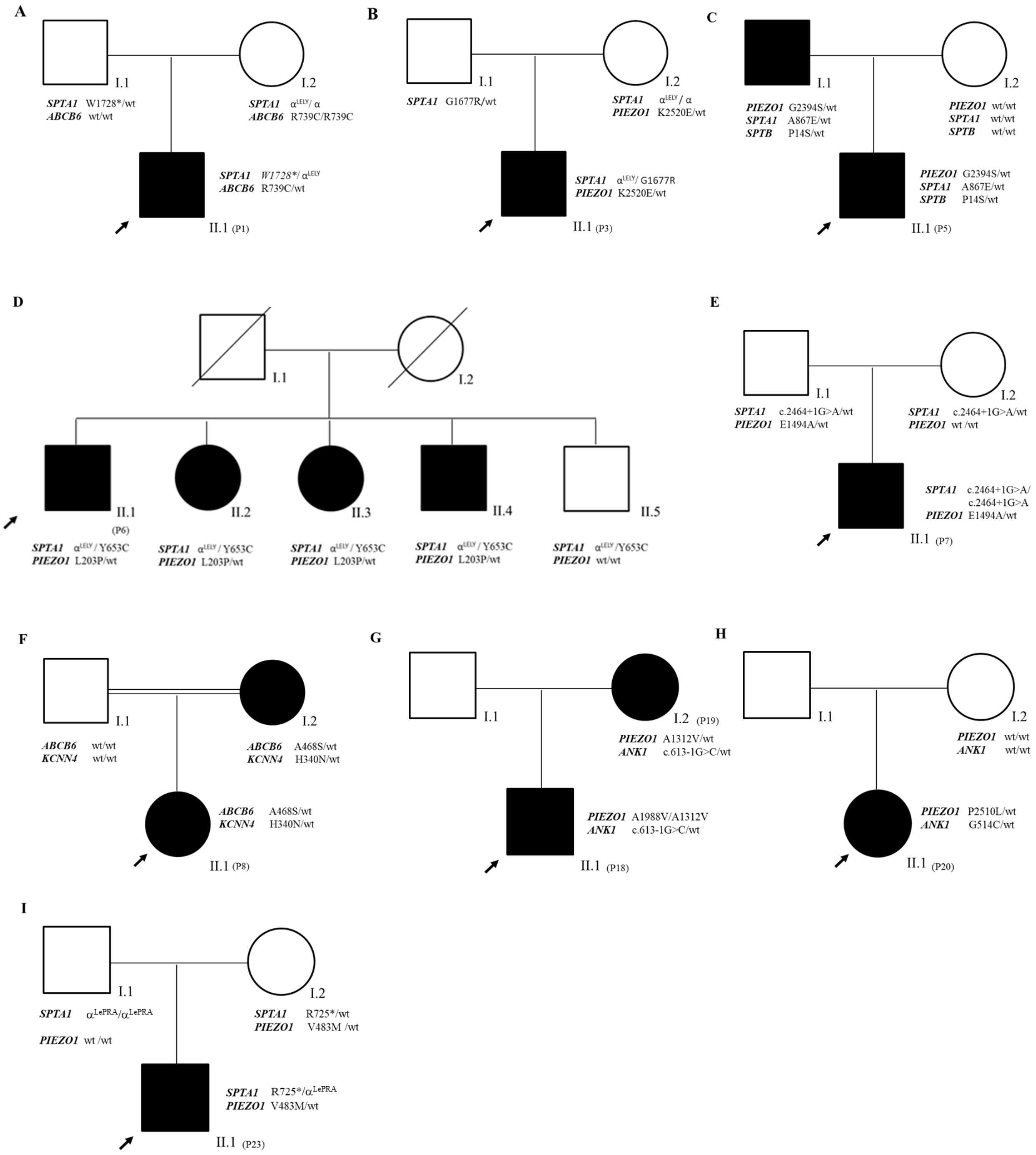

Figure S2. Pedigree of nine probands with digenic inheritance. Males are depicted by squares and females by circles. Black filled symbols indicate individuals with dual/multi-locus genotype. Arrows indicate the probands. Diagonal line indicates death of individual. Double lines between the parents indicate consanguineous marriage. Genotypes are indicated underneath each individual. Inheritance patterns according to the families of the following patients. (**A**) Proband P1. (**B**) Proband P3. (**C**) Proband P5. (**D**) Proband P6. The parents have not been analyzed. (**E**) Proband P7. (**F**) Proband P8. (**G**) Probands P18 and P19. The father has not been analyzed. (**H**) Proband P20. The father has not been analyzed. (**I**) Proband P23. SPTA1-α^{LeLY} (c.6531-12C>T, rs28525570); SPTA1-α^{LePRA} (c.4339-99C>T, rs200830867).

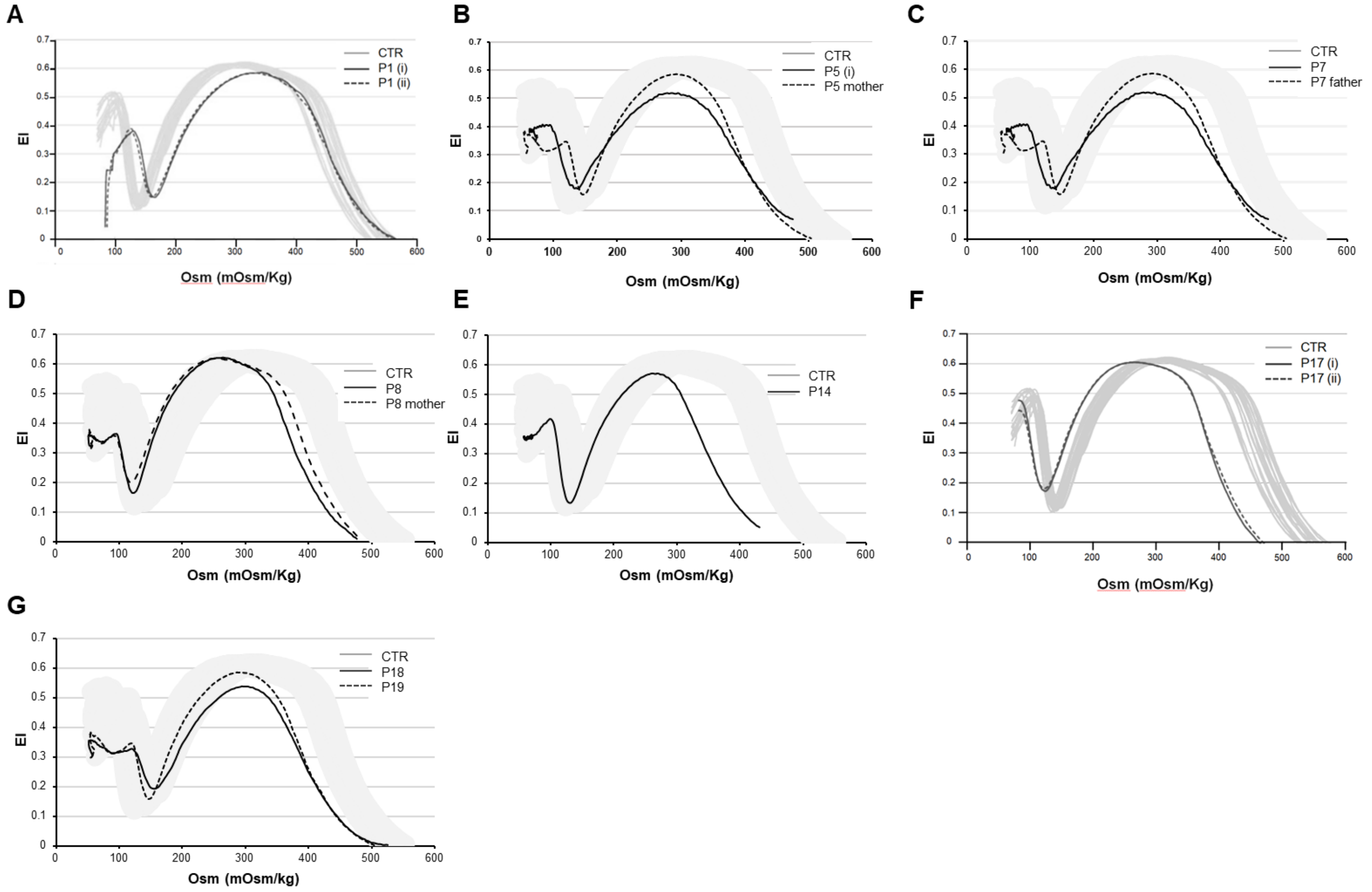

Figure S3. **Osmoscan profiles of some representative dual inheritance cases**. P1 patient (**A**), P5 family (**B**), P7 family (**C**), P8 family (**D**), P14 (**E**), P17 (**F**), P18 and P19 patients (**G**). Continuous lines, curves for the patients; dotted line, curves of parents; shaded areas, curves for the control (CTR) range.

Supplemental Table S1. Additional evidence supporting variant reassessment

Gene	HGVS nomenclature		Other pieces of evidence
	cDNA-level	Protein-level	
ABCB6	c.1361T>C	p.Val454Ala	PS3 (for functional studies performed in Andolfo et al, 2016 PMID: 27151991)
ABCB6	c.1402G>T	p.Ala468Ser	PP1 (for analysis of segregation in the affected family members)
ABCB6	c.1474G>A	p.Ala492Thr	PS3 (for functional studies performed in Fukuda et al 2016, PMID: 27507172, demonstration of impaired ATP binding).<br>BS2 was eliminated because FP is a condition found also in healthy subjects as for example blood donors (Andolfo et al, 2016 PMID: 27151991)
ABCB6	c.1691T>C	p.Met564Thr	-
ABCB6	c.1762G>A	p.Gly588Ser	BS2 was eliminated because FP is a condition found also in healthy subjects as for example blood donors (Andolfo et al, 2016 PMID: 27151991). It is a well-known disease-associated polymorphism with additional supporting functional evidence.
ABCB6	c.2215C>T	p.Arg739Cys	PS3 (for functional studies performed in this study, ionic flux assay, data not shown).
ANK1	c.613-1G>C	-	-
ANK1	c.1540G>T	p.Gly514Cys	-
G6PD	c.1360C>T	p.Arg454Cys	-
KCNN4	c.983A>G	p.His328Arg	PP3 (Multiple lines of computational evidence support a deleterious effect on the gene or gene product)
KCNN4	c.1018C>A	p.His340Asn	PS4 (observed in 3 patients from 2 unrelated families)
LARS2	c.457A>C	p.Asn153His	PP3 (predicted to impair splicing).<br>PP2 (the gnomAD missense Z-Score= 1.33 is greater than 0.647).<br>PP5 (ClinVar classifies this variant as likely pathogenic)
PIEZO1	c.608T>C	p.Leu203Pro	PP1 (Co-segregation with disease in four affected family members).

Supplemental Table S1. (continued)

PIEZO1	c.1001C>T	p.Ala334Val	PM7 (additional moderate pathogenic evidence, left shift of the osmolarity curve).<br>PM1 (Located in a well-established functional domain, transmembrane helical unit 2 (THU2) essential for plasma membrane targeting and inactivation kinetics, Yan Jiang et al 2021 PMID: 33610426).
PIEZO1	c.1447G>A	p.Val483Met	-
PIEZO1	c.1813A>G	p.Met605Val	PM1 (Located in a well-established functional domain, transmembrane helical unit 2 (THU4) essential for mechanosensitivity and Jedi1/2 responses (Yan Jiang et al 2021 PMID: 33610426).<br>PS4 (observed in 3 patients from 2 unrelated families of our DHS registry).
PIEZO1	c.3935C>T	p.Ala1312Val	PS4 (observed in 2 patients from 1 unrelated family of our DHS registry).<br>PM1 (Located in a well-established functional domain, Beam essential for mechanosensitivity; Yoda1, Jedi1/2 responses; inactivation kinetics; margaric acid-mediated inhibition (Yan Jiang et al 2021 PMID: 33610426).<br>PM7 (additional moderate pathogenic evidence, left shift of the osmolarity curve). PP1 (Co-segregation with disease in two affected family members).<br>BS2 was eliminated because DHS is a condition more frequent than expected (for example DHS variants are associated with malaria resistance, Ma et al, 2018).
PIEZO1	c.4481A>C	p.Glu1494Ala	PS4 (observed in 2 patients from 1 unrelated family of our DHS registry).<br>PM7 (additional moderate pathogenic evidence, left shift of the osmolarity curve).<br>PP1 (Co-segregation with disease in two affected family members).
PIEZO1	c.5195C>T	p.Thr1732Met	PM1 (Located in a well-established functional domain, THU8 PM targeting and Yoda1 responses, Yan Jiang et al 2021 PMID: 33610426).<br>PM7 (additional moderate pathogenic evidence, left shift of the osmolarity curve).<br>PP3 (Multiple lines of computational evidence support a deleterious effect on the gene or gene product).<br>BS2 was eliminated because DHS is a condition more frequent than expected (for example DHS variants are associated with malaria resistance, Ma et al, 2018).
PIEZO1	c.5835C>G	p.Phe1945Leu	PM1 (Located in a well-established functional domain, THU9 PM targeting and Yoda1 responses, Yan Jiang et al 2021 PMID: 33610426).<br>PM7 (additional moderate pathogenic evidence, left shift of the osmolarity curve).<br>PP3 (Multiple lines of computational evidence support a deleterious effect on the gene or gene product)
PIEZO1	c.5981C>G	p.Ser1994Cys	PM1 (Located in a well-established functional domain, THU9 PM targeting and Yoda1 responses, Yan Jiang et al 2021 PMID: 33610426).<br>PM5 (Novel missense change at an amino acid residue where a different missense change determined to be pathogenic has been seen before, CM1911799).<br>PP3 (Multiple lines of computational evidence support a deleterious effect on the gene or gene product).

Supplemental Table S1. (continued)

PIEZO1	c.6205G>A	p.Val2069Met	PM1 (Located in a well-established functional domain, THU9 PM targeting and Yoda1 responses, Yan Jiang et al 2021 PMID: 33610426).<br>PM7 (additional moderate pathogenic evidence, left shift of the osmolarity curve).<br>PP3 (Multiple lines of computational evidence support a deleterious effect on the gene or gene product).
PIEZO1	c.6796G>A	p.Val2266Ile	PS4 (observed in 3 patients from 2 unrelated families of our DHS registry).<br>PP1 (Co-segregation with disease in two affected family members).<br>PP3 (Multiple lines of computational evidence support a deleterious effect on the gene or gene product).<br>BS2 was eliminated because DHS is a condition more frequent than expected (for example DHS variants are associated with malaria resistance, Ma et al, 2018).
PIEZO1	c.7180G>A	p.Gly2394Ser	PS4 (observed in 2 patients from 2 unrelated families of our DHS registry).<br>PM7 (additional moderate pathogenic evidence, left shift of the osmolarity curve).<br>BS2 was eliminated because DHS is a condition more frequent than expected (for example DHS variants are associated with malaria resistance, Ma et al, 2018).
PIEZO1	c.7219G>C	p.Glu2407Gln	PS4 (observed in 5 patients from 3 unrelated families of our DHS registry).<br>PM7 (additional moderate pathogenic evidence, left shift of the osmolarity curve).<br>PP1 (Co-segregation with disease in two affected family members)
PIEZO1	c.7367G>A	p.Arg2456His	PS3 (for functional studies performed in Andolfo et al,2013 PMID: 23479567 and in several other studies).
PIEZO1	c.7529C>T	p.Pro2510Leu	PS4 (observed in 4 patients from 4 unrelated families of our DHS registry).<br>PM7 (additional moderate pathogenic evidence, left shift of the osmolarity curve).<br>BS2 was eliminated because DHS is a condition more frequent than expected (for example DHS variants are associated with malaria resistance, Ma et al, 2018).
PIEZO1	c.7558A>G	p.Lys2520Glu	PS4 (observed in 3 patients from 2 unrelated families of our DHS registry).<br>PM1 (Located in a well-established functional domain, CTD Ion permeation and gating; RR sensitivity).<br>PP1 (Co-segregation with disease in two affected family members).<br>BS2 was eliminated because DHS is a condition more frequent than expected (for example DHS variants are associated with malaria resistance, Ma et al, 2018).
PKLR	c.1675C>T	p.Arg559*	-
SEC23B	c.1233+4C>T	-	-
SLC4A1	c.1462G>A	p.Val488Met	-
SLC4A1	c.2608C>T	p.Arg870Trp	PS3 (for functional studies performed in Jarolim et al 1995 PMID: 7530501)

Supplemental Table S1. (continued)

SLC4A1	c.2621T>C	p.Leu874Pro	PM1 (Located in a well-established functional domain that is responsible of the interaction with carbon anhydrase II, Annelies van Vuren et al 2019 PMID: 31723846)
SPTA1	c.460_462dupTTG	p.Leu155dup	PS3 (for functional studies performed in Roux et al 1989. PMID: 2567189; Glele-Kakai et al 1996 PMID: 8857939; Risinger et al 2018 PMID: 30393954).<br>PM1 (Located in a well-established functional domain, self-association domain).
SPTA1	c.1958A>G	p.Tyr653Cys	PS1 (Same amino acid change as a previously established pathogenic variant regardless of nucleotide change, van Vuren et al 2019 PMID: 31723846).<br>PP1 (Co-segregation with disease in four affected family members). BS2 was eliminated because HS is a frequent condition.
SPTA1	c.2173C>T	p.Arg725*	-
SPTA1	c.2464+1G>A	-	-
SPTA1	c.4708G>A	p.Ala1570Thr	-
SPTA1	c.5029G>A	p.Gly1677Arg	PM7 (additional moderate pathogenic evidence, positive EMA test).<br>PP1 (Co-segregation with disease in two affected family members).<br>PP3 (Multiple lines of computational evidence support a deleterious effect on the gene or gene product).
SPTA1	c.5183G>A	p.Trp1728*	-
SPTB	c.40C>T	p.Pro14Ser	PM7 (additional moderate pathogenic evidence, decreased DiMax of osmolarity curve).<br>PP1 (Co-segregation with disease in two affected family members).<br>BS2 was eliminated because HS is a frequent condition.
SPTB	c.871G>A	p.Gly291Ser	PM1 (Located in a well-established functional domain that is responsible of the interaction with protein 4.1R, actin and adducin).<br>PM2 (Absent from controls).<br>PP2 (Missense variant in a gene that has a low rate of benign missense variation and in which missense variants are a common mechanism of disease. The gnomAD missense Z-Score= 0.751 is greater than 0.647).
SPTB	c.1606G>A	p.Asp536Asn	PS3 (or functional studies performed in Russo R et al 2018 PMID: 29396846).

NCBI RefSeq transcript for each gene:
ABCB6, NM_005689; ANK1, NM_000037; G6PD; NM_001042351; KCNN4, NM_002250; LARS2, NM_015340; PIEZO1, NM_001142864; PKLR, NM_000298; SEC23B, NM_006363; SLC4A1, NM_000342; SPTA1, NM_003126; SPTB, NM_001355437.

Supplemental Table S2. Clinical characteristics of the patients with multi-locus inheritance.

Patient ID	Gender	Ethnicity	†Age (years)	RBCs ($\times 10^6/\mu L$)	Hb (g/dL)	Ht (%)	MCV (fL)	MCH (pg)	MCHC (g/dL)	ARC ($\times 10^3/\mu L$)	Tb (mg/dL)	LDH (U/L)	Ferritin (ng/mL)	Number of transfusions	Additional features
P1	m	Swiss	36	2.50	7.8	23	93.0	31.0	34	188	-	-	-	1	Splenomegaly
P2	m	Egyptian	6	2.95	8.5	27	90.5	28.8	31.8	6	0.5	210	1031	12	Consanguineous parents
P3	m	Italian	3	3.29	9.5	27	81.4	28.9	35.5	141	3.9	1012	1239	20	Splenomegaly
P4	f	Italian	1	3.39	12.1	36	104.7	35.7	34.1	-	-	-	-	14*	Fetal ascites
P5	m	Italian	8	3.69	10.1	29	77.8	27.4	35.3	502	3.6	-	113	1	Splenomegaly; jejunal atresia
P6	m	Italian	68	4.90	9.0	28.8	58.1	18.1	31.3	694	0.8	281	747	17	Treated with iron chelators
P7	m	Somali	11	-	10.8	-	80.0	30.0	38.1	14	5.4	-	2000	150	Consanguineous parents; hearing impairment; speech delay; treated with iron chelators; splenectomized
P8	f	Swedish	3	4.90	13.1	39	80.0	27.0	33.8	91	3.5	-	37	3*	Consanguineous parents; fetal ascites; deafness; psychomotor development impairment
P9	f	Italian	37	3.08	10.9	31	100.0	35.4	35.5	-	-	-	511	0	Splenomegaly
P10	m	Turkish	5	3.20	8.0	26	81.0	27.0	33.0	17	2.0	710	4490	50	Splenomegaly; treated with iron chelators
P11	m	Italian	42	4.82	12.3	39	80.9	25.5	31.5	111	1.8	806	76.8	1	Splenomegaly
P12	m	Italian	42	3.88	9.4	30	77.8	24.2	31.1	225	1.6	1361	10.8	0	Splenectomized
P13	m	Italian	19	5.79	17.9	54	94.0	31.0	33.0	-	-	-	838.2	0	Family history of erythrocytosis; hypopigmented macule on the right arm
P14	m	Italian	19	4.50	13.2	39	86.2	29.3	34.0	140	4.6	378	165	-	-
P15	m	Italian	34	4.16	12.4	35	84.1	29.8	35.4	179	3.6	-	-	-	-
P16	m	Italian	5	4.91	11.0	-	72.9	22.1	30.6	-	0.4	260	18	0	-
P17	m	Swiss	18	5.00	16.2	46	92.0	32.0	34.9	193	9.4	143	115	0	Gilbert syndrome; lusoria arteria
P18	m	Italian	18	5.08	15.1	42	81.9	29.7	36.3	148	0.75	-	62.90	1	Splenectomized
P19	f	Italian	51	4	10.4	30.5	76.0	25.9	34.1	75	0.92	-	140	0	Splenectomized

Supplemental Table S2 (continued).

Patient ID	Gender	Ethnicity	†Age (years)	RBCs ($\times10^6/\mu L$)	Hb (g/dL)	Ht (%)	MCV (fL)	MCH (pg)	MCHC (g/dL)	ARC ($\times10^3/\mu L$)	Tb (mg/dL)	LDH (U/L)	Ferritin (ng/mL)	Number of transfusions	Additional features
P20	f	Italian	5	3.31	7.4	21	87.0	22.3	35.4	415	7.1	657	500	23	Gilbert syndrome; splenomegaly

RBC, red blood cells; Hb, hemoglobin; Ht, hematocrit; MCV, mean corpuscular volume; MCH, mean corpuscular hemoglobin; MCHC, mean corpuscular hemoglobin concentration; ARC, absolute reticulocyte count; Tb, total bilirubin; LDH lactate dehydrogenase.
*Intrauterine transfusion, fetal anemia.
†Age at diagnosis;
- data not available
